# PCR detection of malaria parasites and related haemosporidians: the sensitive methodology in determining bird-biting insects

**DOI:** 10.1186/s12936-016-1338-y

**Published:** 2016-05-20

**Authors:** Rasa Bernotienė, Gediminas Valkiūnas

**Affiliations:** Nature Research Centre, Akademijos 2, 08412 Vilnius, Lithuania

**Keywords:** PCR, Blood-sucking insects, *Plasmodium*, *Haemoproteus*, Host preference, Birds

## Abstract

**Background:**

Knowledge about feeding preference of blood-sucking insects is important for the better understanding epidemiology of vector-borne parasitic diseases. Extraction of DNA from blood present in abdomens of engorged insects provides opportunities to identify species of their vertebrate hosts. However, this approach often is insufficiently sensitive due to rapid degeneration of host DNA in midguts. Recent studies indicate that avian malaria parasites (*Plasmodium* spp.) and related haemosporidians (Haemosporida) belonging to *Haemoproteus* can persist both in vectors and resistant blood-sucking insects for several weeks after initial blood meals, and these parasites can be readily detected by polymerase chain reaction (PCR)—based methods. Because avian haemosporidians are cosmopolitan, prevalent and strictly specific to birds, the determination of haemosporidian DNA in blood-sucking dipterans can be used as molecular tags in determining bird-biting insects. This hypothesis was tested by investigation of prevalence of natural haemosporidian infections in wild-caught mosquitoes (Culicidae) and biting midges (Ceratopogonidae: *Culicoides*).

**Results:**

Females of mosquitoes (1072 individuals of three species) and biting midges (300 individuals of three species) were collected in wildlife using simple netting. They were identified and tested individually for the presence of both the haemosporidian parasites and the bird blood using PCR-based methods. Seven different *Haemoproteus* and two *Plasmodium* lineages were detected, with overall infection prevalence of 1.12 and 1.67 % in mosquitoes and biting midges, respectively. In all, the detection rate of avian haemosporidian parasites was three fold higher compared with the detection of avian blood.

**Conclusions:**

Molecular markers of avian malaria parasites and other haemosporidians are recommended for getting additional knowledge about blood-sucking dipterans feeding on bird blood. Many genetic lineages of avian haemosporidians are specific to avian hosts, therefore, the detection of these parasite lineages in blood-sucking insects can indicate their feeding preferences on the level of species or groups of related bird species.

## Background

Many species of blood-sucking insects are involved in transmission of human and animal pathogens [1]. Knowledge on the host preference of vectors is important for better understanding patterns of epidemiology of vector-borne diseases and evolution of host-parasite interactions [2, 3], but remains insufficient, particularly in wildlife. Many species of blood-sucking insects are known to be either ornithophilic or mammalophilic, however, some vector species have broad feeding preference, which often is an adjustment to the availability of their host species [4–6].

Studies on host preferences of blood-sucking insects in wildlife usually are carried out by (1) the polymerase chain reaction (PCR)-based determination of origin of blood meals reported in females [7–11] and (2) the observation insect behaviour in choice situations [12–14]. The latter method is time-consuming. PCR-based methods are relatively easy to use in research if fresh fully-engorged dipteran insects are available for testing. However, the density of resting fully-engorged mosquito females usually is low [15], and the resting sites of *Culicoides* biting midges remain insufficiently described in many species [14]. The percentage of engorged mosquitoes and *Culicoides* spp. females in samples collected by various traps usually is about 0.2–12.5 % [8, 9, 15, 16]. In different studies, the maximum success of PCR-based determination of blood meals in wild-caught fully-engorged insects varied between 17.5 [6] and 92.2 % [10] using vertebrate specific primers. Numerous negative amplifications in these studies likely were due to the DNA degeneration in insect midgut [11]. Kim et al. [17] reported that blood meal identification was successful by PCR-based methods as long as the blood meal residue was distinguishable by eye in the abdomen. Oshaghi et al. [18] determined that the extracted from the blood meal host DNA can be used as a proper template for PCR amplification only 33 h after ingestion.

Mosquitoes, biting midges and blackflies are main vectors of avian haemosporidians, which are obligate heteroxenous blood parasites belonging to the genera *Haemoproteus*, *Plasmodium*, *Leucocytozoon* and some others. Haemosporidian parasites complete sporogony in susceptible dipteran insects after infected blood meals [19–21]. These parasites are cosmopolitan in birds [20]. Additionally, birds are the most diverse and common vertebrate hosts of haemosporidians in all ecosystems. Over 250 species of these parasites have been described and named [20], and many more genetic lineages have been identified and deposited in GenBank. Recent molecular studies indicate that avian haemosporidians can persist both in competent vectors and resistant blood-sucking insects for several weeks after initial infected blood meals. These parasites can be readily detected by PCR-based methods up to 17 days after infected blood meals [22]. That provides opportunities to determine bird-biting insects using haemosporidian molecular markers. Because avian haemosporidians are widespread, prevalent, diverse and strictly specific to birds, we predicted that determination of their DNA lineages can be used as molecular tags in determining bird-biting insects. This hypothesis was tested here by investigation of natural haemosporidian infection in wild-caught mosquitoes (Culicidae) and biting midges (Ceratopogonidae: *Culicoides*) using PCR-based methods.

## Methods

### Study site and dipteran insect collection

Field-work was carried out at the Biological Station of the Zoological Institute of the Russian Academy of Sciences on the Curonian Spit in the Baltic Sea (55º15′ N, 20º86′ E) between 10th and 20th June 2013. Blood-sucking insects were sampled using an entomological net near the Lake Chaika, where density of mosquitoes and biting midges was high. Insects were collected randomly by simple netting from vegetation; they were collected from the net using a pooter and transported to the laboratory within 3 h post sampling. Collected insects were anaesthetized by placing them into a tube covered with a cotton pad wetted with 96 % ethanol. Anaesthetized mosquitoes were sexed, and females were identified using morphological characters [23]. Biting midges were identified according to their wing patterns and other morphological features [24]; their heads were removed to prepare permanent preparations mounted in Euparal for the further confirmation of the identification.

All collected individual insects were fixed separately in tubes containing 96 % ethanol; they were used for PCR analysis. Before DNA extraction all insect females were checked for blood-meal status dividing them into three classes: fed, gravid and empty. Heads of mosquitoes were removed to eliminate the inhibitory effects on PCRs, as recommended in [25]. New needles were used for dissection of each insect to avoid DNA contamination.

### Polymerase chain reaction and sequencing

Total DNA was extracted from each individual insect female using ammonium acetate extraction method [26]. All reactions were performed in 25 µl total volumes, including 50 ng of total genomic DNA template (2 µL), 12.5 µL of DreamTag Master Mix (Thermo Fisher Scientific, Lithuania), 8.5 μL nuclease-free water and 1 μL of each primer.

The quality of extracted DNA was tested using the insect specific primers LCO149 and HCO2198, which amplify a fragment of cytochrome oxidase subunit I (COI) gene of insects’ mitochondrial DNA [27]. The obtained sequences were also used to confirm insect species identification; these sequences were deposited to GenBank. All PCRs with COI primers were positive indicating good quality of extracted DNA.

To determine bird species based on DNA extracted from mosquito blood meals, we applied bird specific primers Avian-3 and Avian-8, which amplify the 473 bp fragment of bird cytochrome *b* (cyt *b*) gene [28]. In parallel, we also used recently developed [29] bird specific primers cytbPF1 and cytbBirdR, which amplify 219 bp fragments of cyt *b* gene. Additional forward primer (cytbBirdF2) was used for semi-nested PCR to amplify the 169 bp fragment of bird cyt *b* gene.

For genetic analysis of haemosporidian parasites, a nested PCR protocol was applied [30]. We amplified a segment of mitochondrial cyt *b* gene using primers HaemNFI and HaemNR3, which are specific to haemosporidians belonging to *Haemoproteu*s, *Plasmodium* and *Leucocytozoon* parasites. For the second PCR, we used primers HAEMF and HAEMR2, which are specific to *Haemoproteus* and *Plasmodium* spp. and amplify 479 bp fragments of cyt *b* gene. All samples were tested for bird and parasite DNA in parallel. In all PCRs, one negative control (nuclease-free water) and one positive control (one thorax of *Ochlerotatus cantans* mosquito 7 days after the experimental infection with *Haemoproteus tartakovskyi*, in the case of parasite testing and a siskin’s (*Carduelis spinus*) blood sample in case of avian blood testing) were used per every 24 samples to control for false amplifications. No case of false positive or negative amplification was found. Thermal conditions of COI PCR were as described by Day et al. [31], conditions of other PCR followed the protocols mentioned in the references cited.

All positive amplifications were evaluated by running 2 μl of the final PCR product on a 2 % agarose gel; they were sequenced in order to determine cyt *b* lineages of the detected parasites or to detect bird species from blood meals. DNA fragments were sequenced with corresponding primers twice for both strands. Dye terminator cycling sequencing (BigDye) was used and loaded on an ABI PRISM™ 3100 capillary sequencing robot (Applied Biosystems, Foster City, California). Good quality sequences were obtained; they were edited and aligned using BioEdit (Version 7.0.9.0).

### Data analysis

The “Basic Local Alignment Search Tool” (National Centre for Biotechnology Information) was used to determine genetic lineages of detected avian blood parasites or to determine species of birds. The analyses were carried out using “Statistica 7” package. Percentages of infected insects were compared by Yates corrected χ2 test. A *P* value of 0.05 or less was considered significant.

## Results

In all, 1372 females (1072 mosquitoes and 300 *Culicoides* biting midges) were sampled and investigated for the presence of both bird blood and avian haemosporidian parasites using PCR-based methods (Table 1). Three mosquito species were sampled. *Ochlerotatuscantans* was the most abundant mosquito species (69.2 % of all collected mosquitoes); *Ochlerotatuscataphylla* and *Ochlerotatussticticus* represented 22.4 and 8.4 % of all collected mosquitoes, respectively. Three *Culicoides* species were collected and examined. These were *Culicoides impunctatus* (89.0 % of all sampled midges), *Culicoides punctatus* (9.3 %) and *Culicoides pictipennis* (1.7 %). Obtained COI sequences of investigated insects conform for 99–100 % with sequences of corresponding species from GenBank [KJ627800, KX064673–KX064677]. The great majority of collected insects were classified as empty (96.4 %). Gravid and fed females represented 3.1 and 0.5 %, respectively.

**Table 1:** Sensitivity of PCR-based detection of bird blood and haemosporidian lineages in the same wild-caught blood-sucking dipteran insects

Dipteran insect family and species	No. examined	No. positive for bird blood	No. positive for parasites	Difference, *P* value	Parasite lineage
Mosquitoes Culicidae					
*Ochlerotatus cantans*	742	2 (0.27)^a^	8 (1.08)	0.113	pSGS1, pGRW11, hSISKIN1, hDELURB1, hSW1, hRB1, hRBS2
*Oc. cataphyla*	240	1 (0.42)	3 (1.25)	0.616	hRBS2, hSFC1
*Oc. sticticus*	90	0 (0)	1 (1.11)	0.316	hSISKIN1
Total mosquitoes	1072	3 (0.28)	12 (1.12)	*0.038*	Eight lineages reported
Biting midges Ceratopogonidae					
*Culicoides impunctatus*	267	2 (0.75)	3 (1.12)	0.472	pSGS1, hTURDUS2, hSISKIN1
*C. punctatus*	28	1 (3.57)	1 (3.57)	1.000	hTURDUS2
*C. pictipennis*	5	0 (0)	1 (20.0)	0.292	hTURDUS2
Total biting midges	300	3 (1.00)	5 (1.67)	0.722	Three lineages reported
Grand total	1372	6 (0.44)	17 (1.24)	*0.036*	Nine lineages reported

^a^Percentage is given in parenthesis. Significant differences are given in italic font

Seven different *Haemoproteus* spp. (hSISKIN1, hDELURB1, hSW1, hRB1, hRBS2, hSFC1, hTURDUS2 [GenBank accessions KC559436, KC818455, KJ559439, KJ627802, KR049256–KR049262]) and two *Plasmodium* spp. (pSGS1, pGRW11 [GenBank accessions KR049253–KR049255]) cyt *b* genetic lineages were detected, with overall infection prevalence of 1.12 % in mosquitoes and 1.67 % in biting midges (Table 1).

Blood of three bird species (*Ficedula hypoleuca, Carduelis spinus* and *Parus major*) was detected in 0.44 % of examined insects; it was identified in three individual mosquitoes and three individual biting midges using primers cytbBirdR/cytbBirdF2 (Table 1). Obtained DNA sequences conform for 98–100 % with the sequences of corresponding bird species from GenBank [accessions KP794613–KP794615]. The efficacy of primers Avian-3/Avian-8 was similar: bird blood was detected in 0.36 % of examined insects (in three individual mosquitoes and two individual biting midges). Both sets of bird specific primers amplified DNA from the same samples, except one sample of *C. impunctatus,* in which one positive amplification was obtained using cytbBirdR/cytbBirdF2 primers, but not with Avian-3/Avian-8 primers. Differences between sensitivity of different primers in detection of bird blood were insignificant (*P* = 0.76).

Overall, the detection rate of avian haemosporidian parasites was three-fold higher (*P* < 0.05) comparing with the detection rate of avian blood in same samples of investigated blood-sucking insects (Table 1).

## Discussion

The key result of this study is that molecular markers of avian haemosporidians can be readily used for determining bird-biting dipteran insects. Application of this methodology in parallel with detection of bird blood in engorged insects provides much additional information about feeding preference of blood-sucking dipterans in wildlife.

It is difficult to collect large samples of engorged blood-sucking dipterans for determination of origin of their blood meals [9, 15, 16]. Simple netting from low vegetation was used to collect mosquitoes and biting midges in this study. Blood-sucking insects are also often collected using animal baits and light or ultraviolet traps in wildlife [13]. These sampling methods provide opportunities to collect the actively flying part of dipteran populations, particularly females, which are host seeking insects. Selective collection of engorged females directly in their resting sites is time consuming, particularly because of insufficient knowledge about their resting places, which might differ even for the same insect species at different study sites [14]. Proportion of engorged mosquitoes and *Culicoides* spp. usually varies markedly in different studies, and it has been reported not to exceed 12.5 % of all sampled females [8–10, 15]. Even if sufficient number of engorged insects is collected, the probability to obtain positive PCR signals usually is not high using primers specific for vertebrate host DNA. The success of PCR amplification of host DNA in engorged mosquitoes using vertebrate specific primers was 17.5 % in Portugal [6]. Bartsch et al. [8], Lassen et al. [9] and Ninio et al. [32] reported 65, 76 and 91 % of positive PCR amplifications of host DNA in fully engorged biting midges in Germany, Denmark and France, respectively. Negative PCR-based results are usually explained by the low blood meal volumes or the degeneration of host DNA due to blood digestion in the midguts [11]. Additionally, there is a significant negative relationship between the time after blood ingestion and the success of PCR-based analysis [18, 33]. According to Ejiri et al. [28] the success of blood meal identification using PCR is 100 % in fresh full-fed mosquitoes, 75 %—in partially fed, 56 %—in half-gravid and only 38 %—in gravid females.

Molecular markers of avian haemosporidian parasites are suggested to use for determining bird-biting insects (Table 1). The prevalence of haemosporidian infections is high in many bird species worldwide, with the overall prevalence exceeding 40 % and reaching even 80–100 % in many bird populations during the breeding season in Europe [20, 34]. Blood-sucking insects gain these parasites only during infected blood meals on birds. Alive sporozoites of avian malaria parasites and other haemosporidians have been reported in *Culex* spp. and *Culicoides* spp. for approximately one month after initial infected blood meal [19, 20]. Additionally, haemosporidian parasites can persist even in resistant blood-sucking insects without DNA degeneration for several weeks after infected blood meals [22]. That provides opportunities to identify bird-biting insects using molecular markers, which target haemosporidian parasite DNA. For example, haemosporidian parasites have been reported between 0.4 and 33.7 % of pools of wild-caught dipteran females tested by PCR at different study sites [6, 35]. We determined avian haemosporidian parasites in 1.24 % of all tested insects (Table 1). Martínez-de la Puente et al. [36] determined 6.4 % of collected *Culicoides* spp. to be positive for haemosporidian infection in Spain. Ejiri et al. [28] reported 14.6 % of tested *Culex pipiens* mosquitoes infected by these parasites in Japan. In Switzerland, 6.6 % of *Culex pipiens* females were PCR positive for avian malaria infection [37]. Inci et al. [38] found DNA of avian haemosporidian parasites in 9.9 % of mosquito pools in Turkey. Thus, it is relatively easy to detect haemosporidian parasites in blood-sucking insects using PCR-based diagnostics. Additionally, it is known that chronically infected vertebrate hosts attract significantly more vectors than uninfected ones [39, 40], and this fact further increases the efficiency of parasite detection compared with the host blood detection in insect. Because avian haemosporidians are strictly specific to birds [2, 20], the reports of their lineages in dipterans indicate their ornithophily (Table 1).

This study detected the widespread bird malaria parasite *Plasmodium relictum* (lineages pSGS1 and pGRW11) in *Ochlerotatus cantans* mosquitoes, and the lineage pSGS1 was also detected in *Culicoides impunctatus*, indicating that these insects naturally feed on birds. Formerly, both insect species were considered to be mainly mammalophilic [4, 41]. Interestingly, Santiago–Alarcon et al. [3], Ferraguti et al. [42], Martínez-de la Puente et al. [36] and this study detected avian *Plasmodium* lineages in biting midges, the insects which do not transmit these parasites [2], indicating feeding preference of *Culicoides* midges and possible abortive sporogonic development in non-susceptible insects [22]. In this study, six different *Haemoproteus* lineages were detected in mosquitoes, which also do not transmit these parasites. These lineages are: hSISKIN1 (*Haemoproteus tartakovskyi),* hRB1 and hRBS2 (*Haemoproteus lanii*), hSW1 (*Haemoproteus belopolskyi*), hDELURB1 (*Haemoproteushirundinis*), and hSFC1 (*Haemoproteus balmorali*) (Table 1). Mosquitoes are non-competent hosts of *Haemoproteus* spp., but are exposed to these parasites during infected blood meals, and the parasite’s DNA can be detected in mosquitoes even several weeks after initial blood meals [22] providing opportunities to identify bird-biting insects.

It worth noting that some lineages of haemosporidian species are known to complete life cycle and produce gametocytes (the only infective stage for vectors) in certain avian hosts. That is particularly true for some *Haemoproteus* parasites [20, 43]. Detection of such parasite lineages in blood-sucking insects might indicate their feeding preference on the level of species or groups of relative bird species. For example, the lineage hSISKIN1 of *H. tartakovskyi* has been reported to complete development and produce gametocytes only in siskins and crossbills (*Loxia curvirostra*); the lineage hSW1 of *H. belopolskyi* and the lineages hRB1 and hRBS2 of *H. lanii* complete development only in *Acrocephalus* and *Lanius* birds, respectively (see Table 2). Gametocytes of the lineage hTURDUS2 of *H. minutus* and the lineage hALARV of *H. tartakovskyi* were reported only in blackbirds (*Turdus merula*) and skylarks (*Alauda arvensis*), respectively [50, 70, 71]. The reports of these *Haemoproteus* lineages in blood-sucking insects (Table 1) indicate that they likely naturally take blood meals on corresponding bird species.

**Table 2:** Occurrence of detected *Haemoproteus* parasites and their cytochrome *b* lineages in European birds

Parasite species	Genetic lineage	Vertebrate hosts positive by		References
		PCR-based detection	Microscopic examination	
*H balmorali*	hSFC1	*Muscicapa striata; Cyanistes caerulens*	*Muscicapa striata*	[44–49, 50 ^a^]
*H. belopolskyi*	hSW1	*Acrocephalus schoenobaenus; Acrocephalus palustris; Acrocephalus scirpaceus*	*Acrocephalus schoenobaenus; Acrocephalus palustris*	[45, 51–53, 54 ^a^, 55]
*H. hirundinis*	hDELURB1	*Delichon urbicum; Hirundo rustica*	*Delichon urbicum*	[45, 56–58, 59 ^a^]
*H. lanii*	hRB1	*Emberiza schoeniclus; Lanius collurio*	*Lanius collurio*	[45, 53, 60 ^a^, 61 ^a^]
	hRBS2	*Lanius collurio*	*Lanius collurio*	[45, 46, 62 ^a^]
*H. minutus*	hTURDUS2	*Acrocephalus scirpaceus; Bolborhynchus lineola; Cyanistes caeruleus; Emberiza schoeniclus; Eritacus rubecula; Garulus glandarius; Hippolais icterina; Lanius collurio; Panurus biarmicus; Turdus merula; Turdus torquatus*	*Turdus* *merula*	[44–46, 49, 50 ^a^, 53, 60 ^a^, 63–67]
*H. tartakovskyi*	hSISKIN1	*Carduelis spinus; Loxia curvirostra; Carpodacus erythrinus*	*Carduelis spinus; Loxia curvirostra*	[22 ^a^, 50 ^a^, 53, 60, 61 ^a^, 68, 69 ^a^]

^a^Studies reporting presence of gametocytes in the blood of birds

However, the information about feeding preference of blood-sucking insects on the level of bird species should be analysed with caution using parasitology data. Public databases [72] and the GenBank, contain information about reports of some parasite lineages in unusual avian hosts, in which gametocytes of corresponding lineages have not been reported by microscopic methods [20], and solely PCR-based diagnostic data are available (Table 2). The number of such reports is increasing in MalAvi database [72], and they likely are due to abortive development of parasites, which do not produce gametocytes and cannot infect blood-sucking insects [59, 73]. It is important to note that PCR-based method is a sensitive tool for detection of haemosporidian parasites however, it only requires presence of parasite DNA, so can overestimate host range when such DNA is detected from non-competent host species. In other words, solely PCR-based record of a lineage in a host species is not enough evidence of the species being a competent host, i.e. the host in which haemosporidian gametocytes develop. Table 2 provides references of studies, which detected gametocytes in birds for the lineages reported in this study. Vectors can be infected and parasite can develop (completely or abortively) in insects only after blood meal on birds with gametocytes in the blood [20]. Other haemosporidian stages, which might be present in the circulation (remnants of tissue meronts or sporozoites) and provide templates for PCR amplification, are digested in blood-sucking insects [19, 20]. There are increasing evidences that haemosporidian infections in non-competent hosts result in abortive development, and gametocytes are not produced [22, 67, 74]. Because such DNA may leak into the blood, it is likely that the host range across bird families or genera for many parasite lineages reported by PCR is broader than the ranges of their competent hosts (Table 2). That should be taken in consideration when using the methodology described in this study: positive PCR signals in blood-sucking insects likely indicate that they were fed on competent hosts of corresponding parasite lineages. *Plasmodium* parasites are less specific for vertebrate hosts [19], and reports of their lineages in blood-sucking insects can be used mainly for indication of feeding on the level of class Aves.

Due to complex patterns of transmission of pathogens [75], the determination of ornithophilic blood-sucking insects is important epidemiologically, including the epidemiology of human vector-borne diseases, because the same vector can take blood meal from different hosts and can serve as bridge vector of the disease from birds to humans. Some mosquitoes and biting midges are opportunistic in trait of host choice [9], but others are host-specific and prefer to take blood meals either on mammals or birds [15]. Temporal and spatial variation in host availability might be important in host selection by blood-sucking insects [14]. For example, many *Culex* species prefer to feed on birds [14], however, this host-insect interaction is not strict. The abundance of birds often changes due to seasonal and other migrations at many sites throughout the year, and *Culex* species can readily switch from birds to humans when the availability of birds declines [76]. For example, many *Culex* species prefer to take blood meals on the American robin (*Turdus migratorius*) in the Nearctic. However, when the density of this bird population decreases during migrations, *Culex* mosquitoes shift from this preferred avian host to humans, thereby increasing a probability of West Nile virus transmission to humans [77]. More information is needed about feeding specialization of blood-sucking dipterans. Detection of haemosporidian lineages in dipterans contributes to accumulation of such knowledge.

It worth mentioning that the same insect can be infected with several species of haemosporidian parasites due to the blood meal on birds with co-infection or the “contamination” by any subsequent blood meal on different species of avian hosts. This situation makes analysis of possible host species of blood-sucking insects more difficult. Recent experimental study [78] shows that application of several primers in parallel markedly increase detectability of haemosporidian parasite co-infections, and this method worth using in vector research as well.

Haemosporidian parasites are particularly diverse in countries with warm climates [19]. In Europe and other countries with temperate or cold climates, haemosporidian parasites are widespread in birds, but usually are rare or absent in mammals and reptiles. However, many groups of terrestrial vertebrates are parasitized by haemosporidians in tropical countries [19, 20, 79]. It is probable that the methodology developed in this study can be applied for determining reptile, bird and mammal feeding blood-sucking insects in countries where active transmission of these parasites occurs. Wildlife haemosporidians can be used as biological markers for determining feeding preference of blood-sucking insects even more effectively in tropical and subtropical regions where these pathogens inhabit not only birds, as mainly is the case in northern Holarctic [2, 20], but also many species of reptiles and mammals [19, 79].

## Conclusions

This study shows that PCR-based detection of lineages of avian haemosporidian parasites is useful methodology for determining ornithophilic blood-sucking insects. If applied together with molecular markers targeting bird DNA, this methodology increases probability of determining host preference of dipteran blood-sucking insects. Molecular markers of avian malaria parasites and other haemosporidians as additional biological tags in determining links between blood-sucking insects and their avian hosts in wildlife are recommended.
